# The Fast Cognitive Evaluation (FaCE): a screening tool to detect cognitive impairment in patients with cancer

**DOI:** 10.1186/s12885-022-10470-1

**Published:** 2023-01-09

**Authors:** Amel Baghdadli, Giovanni G. Arcuri, Clarence G. Green, Lynn R. Gauthier, Pierre Gagnon, Bruno Gagnon

**Affiliations:** 1 CHU de Québec-Université Laval Research Centre, Québec City, QC Canada; 2 Montreal University Hospital Center (CHUM) Research Centre, Montreal, QC Canada; 3 Faculty of Medicine and Health Sciences, School of Physical and Occupational Therapy, McGill University, Montreal, QC Canada; 4 Federation University Australia, Berwick, Melbourne, Vic Australia; 5 Équipe de recherche Michel-Sarrazin en oncologie psychosociale et soins palliatifs, Québec City, QC Canada; 6 Department of Family and Emergency Medicine, Faculty of Medicine, Université Laval, Québec City, QC Canada; 7 Université Laval Cancer Research Centre, Québec City, QC Canada; 8 Réseau Québécois de recherche en soins palliatifs et de fin de vie du FRQS (RQSPAL), Québec City, QC Canada; 9 VITAM-Centre de recherche en Santé Durable, Université Laval, Québec City, QC Canada; 10 Department of Psychiatry and Neurosciences, Université Laval, Québec City, QC Canada

**Keywords:** Chemo brain, Brain fog, Cognition, Cancer, Mild cognitive impairment, Memory

## Abstract

Cancer-related cognitive impairment (CRCI) is one of the most concerning conditions experienced by patients living with cancer and has a major impact on their quality of life. Available cognitive assessment tools are too time consuming for day-to-day clinical setting assessments. Importantly, although shorter, screening tools such as the Montreal Cognitive Assessment or the Mini-Mental State Evaluation have demonstrated a ceiling effect in persons with cancer, and thus fail to detect subtle cognitive changes expected in patients with CRCI. This study addresses this lack of cognitive screening tools by developing a novel tool, the Fast Cognitive Evaluation (FaCE).

A population of 245 patients with 11 types of cancer at different illness and treatment time-points was enrolled for the analysis. FaCE was developed using Rasch Measurement Theory, a model that establishes the conditions for a measurement tool to be considered a rating scale.

FaCE shows excellent psychometric properties. The population size was large enough to test the set of items (item-reliability-index=0.96). Person-reliability (0.65) and person-separation (1.37) indexes indicate excellent internal consistency. FaCE’s scale is accurate (reliable) with high discriminant ability between cognitive levels. Within the average testing time of five minutes, FaCE assesses the main cognitive domains affected in CRCI.

FaCE is a rapid, reliable, and sensitive tool for detecting even minimal cognitive changes over time. This can contribute to early and appropriate interventions for better quality of life in patients with CRCI. In addition, FaCE could be used as a measurement tool in research exploring cognitive disorders in cancer survivors.

**Supplementary Information:**

The online version contains supplementary material available at 10.1186/s12885-022-10470-1.

## Introduction

In 2020, the Canadian Cancer Society estimated that 225,800 Canadians were diagnosed with cancer [1]. It is reported that the 5- and 10-year average survival rates for persons with cancer, all cancers combined, are 63% and 57%, respectively [1]. Although survival rates have risen, cancer is often accompanied by secondary symptomatology from the malignancy itself or its treatments, months, or years into remission. Cancer-related cognitive impairment (CRCI) is amongst the most troublesome, as it not only affects patients’ quality of life, but also significantly limits their daily and social functioning including their return to active professional life [2–5]. CRCI was first recognized by cancer survivors themselves [6] and is estimated to affect 75% of patients during treatment [7] and approximately 25-35% years after treatment [3, 8]. Unfortunately, CRCI remains underdiagnosed and challenging to treat [5, 9]. CRCI is described as a syndrome presenting as a combination of difficulties with working memory, decreased concentration, attention deficits, reduced verbal fluency, and impaired executive function [9].

The first step in treating any condition, including CRCI, is the ability to detect its occurrence. Presently, there is a lack of cognitive screening tools applicable to CRCI adapted to the clinical setting and none are specific to this population, nor can they detect the subtle cognitive changes seen in CRCI, detect them early, and evaluate them over time (before, during, and after the course of treatment). Currently available cognitive assessment tests such as the Repeatable Battery for the Assessment of Neuropsychological status [10] and the High Sensitivity Cognitive Screen [11] are too time-intensive to be used in everyday clinical practice [12]. The Mini-Mental State Examination (MMSE) [13] and the Montreal Cognitive Assessment (MoCA) [14] have been used for decades as cognitive screening tools in the geriatric population for suspected dementia and more recently, in other populations. Although these tools are more time-efficient, our group has previously demonstrated that the MoCA, when administered to persons with cancer, presents a noticeable ceiling effect, meaning that it cannot detect subtle cognitive deficits [4, 15]. These results confirmed the conclusions of other authors [16, 17]. This limitation is of great importance since patients with cancer are expected to show better cognitive ability than patients with dementia as they do not present with widespread organic brain degeneration [18, 19].

This present study addresses the current gap of cognitive assessment tools specific to cancer survivors as defined by the American Cancer Society [20] by presenting a novel tool developed specifically with persons with cancer. This tool is rapid, valid, reliable, specific, and sensitive in detecting even minimal cognitive changes.

### Methodology

First, a French cognitive assessment tool, the Fast Cognitive Screen (FCS) (Appendix 2), was developed using our previous research findings with the MoCA [15] and the recommendations and systematic reviews published by various expert groups [9, 21–23]. Second, by applying Rasch Measurement Theory (RMT) to the FCS, a novel tool with excellent psychometric properties was developed which we called the Fast Cognitive Evaluation (FaCE).

The study was approved the Research Ethics Board of the CHU de Québec (2014-1209; A13-05-1209; and 2017-3312).

### Population

Patients diagnosed with cancer at any stage, before chemotherapy, currently or previously treated, were recruited from outpatient clinics of the CHU de Québec-Université Laval, Québec City, QC, Canada, between August 2013 and August 2018 by contacting patients with the permission of the treating team, and a portion of women with breast cancer before chemotherapy within a study on chemotherapy-induced peripheral neuropathy [24]. Patients with brain tumors or brain metastases, history of cerebrovascular disease, psychiatric illness, or dementia were excluded. Informed consent was obtained for all participants.

### Tool development, data collection and testing

The FCS included questions assessing cognitive domains commonly impacted by CRCI such as: orientation in time and space, visuospatial abilities (copying a cube, drawing a clock), executive functioning (a Trial Test (A-H; 1-8)), attention (5 sequential subtractions, repetition of 4 numbers forward and 3-7 numbers backward). Moreover, the difficulty levels of working memory [25] tasks were increased to address the ceiling effects previously highlighted [15] (repeating 7 words twice instead of only 5 words (as in the MoCA)), verbal fluency (naming as many fruits and vegetables as possible in one minute), and delayed recall (recalling the 7 previously stated words).

Socio-demographic and medical information were collected through a structured questionnaire and medical chart review. The FCS was then administered to all participants by trained evaluators. All four evaluators received standardized training including a minimum of two shadowing sessions with skilled evaluators and an instruction booklet.

### Rasch measurement theory analysis

Rasch Measurement Theory [26] was then applied to the FCS to develop the FaCE tool. RMT is based on the premise that the probability of each response is correlated with the interaction between the difficulty level of an item and the ability level of the examinee: people with higher abilities are more likely to correctly answer more difficult items than people with lower ability levels. It depicts the conditions to be satisfied for a measurement tool to be considered a rating scale, that is, with proportional level of difficulties [27]. Rasch analyses were conducted using Winsteps version 4.1.5 statistical software [28].

Test-of-fit statistics (infit and outfit residuals) between 0.5 and 1.5 were considered suitable fit [29]. Differential item functioning (DIF) analyses for age, sex, education, cancer stage, chemotherapy and radiotherapy status, comorbidities, and interviewers were also conducted to determine response patterns to items which did not fall within the RMT predictions [30]. A principal components analysis (PCA) of residuals was performed to determine if the FCS is unidimensional, meaning that it evaluates a single latent trait (i.e., cognition) [31]. Reckase’s criterion proposes that unidimensionality is achieved when the variance explained by a measure is at least 20 % [32] and, as proposed by Linacre, an eigenvalue of at least 3 in each contrast was used as a cut-point to determine the need for further investigation of the items included in the identified cluster [31]. To support the scale’s reliability (internal consistency), separation statistics above 2 and reliability above 0.8 were used [33].

### Development of the Fast Cognitive Evaluation tool based on the Fast Cognitive Screen

The FCS underwent two subsequent Rasch analyses to improve its measurement performance and select the minimal and most reliable set of items. After each analysis, items were removed if they did not fit the model or prevented the FCS from meeting the *a priori* requirements to be considered an ideal rating scale.The tool demonstrated excellent psychometric properties after two rounds of analysis. . An Analysis of Variance (ANOVA) was performed to test the effect of both the time needed to complete the FCS and which evaluator conducted the test on the score. The optimized version of the FCS was then renamed the Fast Cognitive Evaluation (FaCE) (Appendix 1).

### Development of a second French and two English Versions of the Fast Cognitive Evaluation

A second equivalent French version and two additional English versions of FaCE were developed by a linguistic specialist by carefully matching French-English word-stimuli on a set of psycholinguistic variables, including word frequency [34], word/syllable length [35], imageability [36], age of acquisition [37] and reaction time norms [38]. One pair of French/English versions of FaCE measures semantic verbal fluency through animal naming, while the other uses fruits/vegetables, as these are stable across French and English in terms of the number of items typically named in 60 seconds [39, 40]. Full details are provided in Appendix 3.

## Results

### Population

During the study period, 246 participants met eligibility criteria. One was excluded because of major discrepancies in responses. The study population included 67% women, from 26 to 95 years old, where approximately half (48%) held a university degree. The study participants had 11 different cancer types, with 46% of participants having breast cancer, as expected since one of our largest recruitment sites was a breast cancer clinic. Table 1 summarizes population characteristics. FaCE was administered at different timepoints, before (44%), during (35%), and after completing chemotherapy (21%).

**Table 1:** Participants’ characteristics

**Participants' characteristics (*N*=245)**	***N* (%)**
**Age (years)**	
Mean (SD): 59.8 (13.06) Range [Min-Max]: [28-95]	
< 70	185 (76)
≥70	60 (24)
**Female**	165 (67)
**Highest education level**^**c**^	
No university degree	126 (52)
University degree	118 (48)
**Type of cancer**^**a**^	
Breast	113 (46)
Digestive tract	41 (17)
Prostatic	27 (11)
Gynecologic	23 (9)
Lymphatic	16 (7)
Lung	7 (3)
Other (Thyroid, skin, head, neck, hematologic, etc.)	26 (11)
**Chemotherapy status**^**d**^	
Before chemotherapy or none scheduled	92 (44)
During chemotherapy^b^	73 (35)
After chemotherapy	44 (21)

*SD* Standard deviation

^a^One participant may have had more than one cancer

^b^"During chemotherapy" corresponds to a period from the first chemotherapy treatment to 6 months after the last chemotherapy treatment; beyond this period is the time referred to as "After chemotherapy"

^c^1 missing data

^d^36 missing data

### Results of rasch analyses of the FCS to develop FaCE

The FCS went from 55 items assessing 6 cognitive dimensions to FaCE with 31 items assessing 5 dimensions. After the first Rasch analysis, orientation questions, clock drawing, and backward and forward repetition of a series of numbers were removed because of weak correlation between the observed and expected scores (0 < Point measure correlation < 0.1). It is estimated that FaCE takes an average of 5 minutes to administer.

**Fig. 1:**
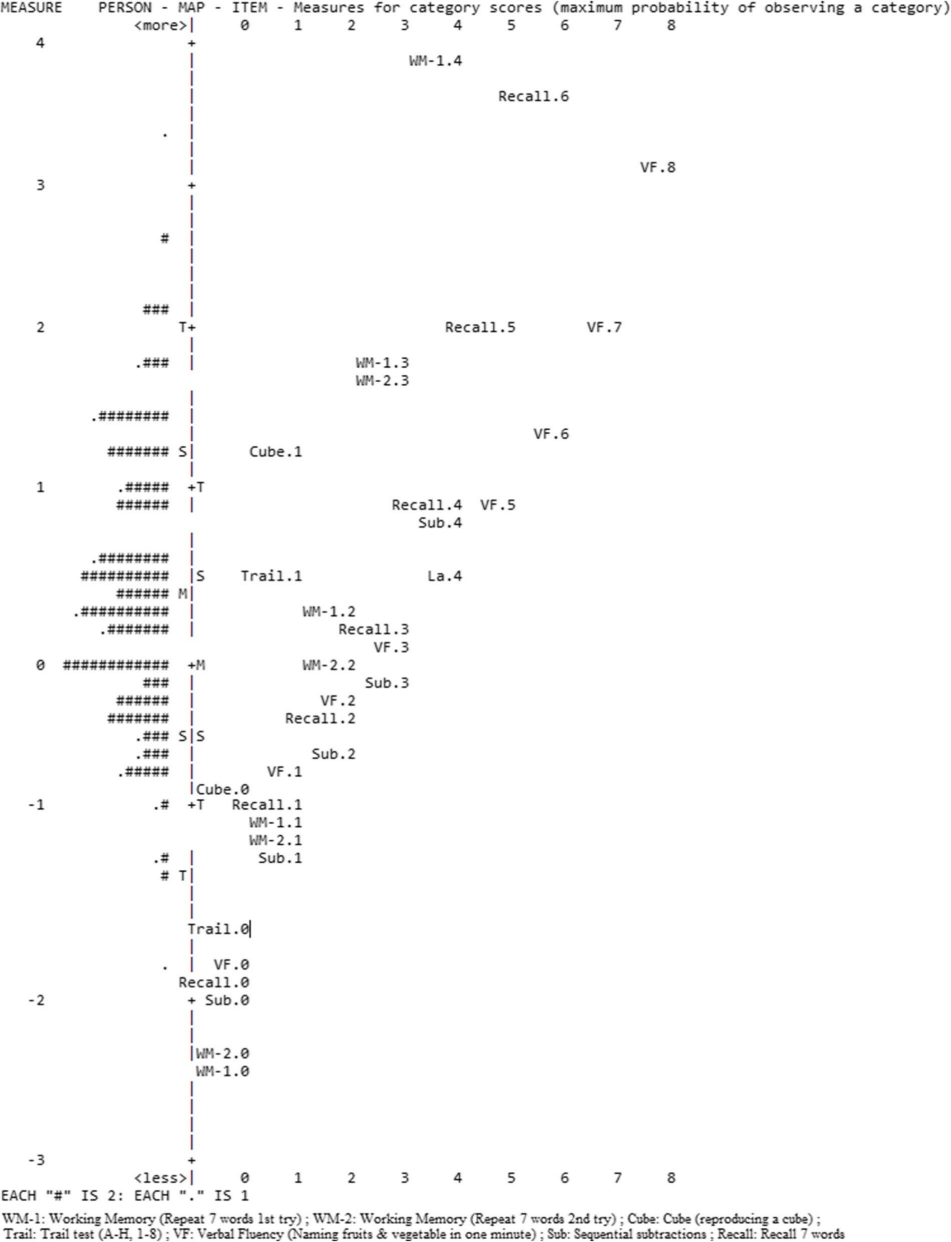
Person-Item Map: Measures for category scores (maximum probability of observing a category). In the Person-Item Map the items are positioned at their highest level of probability of reaching a pre-defined category on the rating scale (expected score on the item; for example, VF.7 is positioned at 2 Logit). The vertical axis is on a Logit scale; to its left is the distribution of persons (by ‘#’ for 2 persons or ‘.’ for 1 person) and to its right is the items hierarchy (where each item is abbreviated, e.g., VF: Verbal Fluency). The persons are arranged by increasing cognitive capacity from bottom to top; the items by increasing difficulty. The number following each abbreviation corresponds to the scoring category used for the analysis, which could correspond to more than 1 possible answer (e.g. VF.6 corresponds to naming 25 to 26 fruits & vegetables in one minute). ‘M’: Mean cognitive capacity. ‘+M’: Mean probability of successfully answering the items. ‘S’ and ‘T’: First and second standard deviations of mean cognitive capacity, respectively. ‘+S’ and ‘+T’: First and second standard deviations of probability of successfully answering the items, respectively

As shown in Table 2, the Rasch analysis conducted on FaCE showed no gaps in the scale, nor floor or ceiling effects, since no participant scored 0 or 27, respectively. All items adequately fit the model (0.5 < Outfit MNSQ < 1.5). Figure 1 presents a visual conceptualization of FaCE and its measures. The vertical axis is on a *Logit* scale, which is a mathematical conversion of the probability (0 to 1) of an individual obtaining a particular score. To the left of the vertical axis is the distribution of persons arranged by increasing cognitive capacity from bottom to top. The letter ‘M’ represents the mean cognitive capacity (0.3 *Logit*). To the right of the vertical axis are the questionnaire items, arranged by increasing difficulty from bottom to top. The letter ‘+M’ represents the mean probability of successfully answering the items (0 *Logit*). According to RMT, the results in Fig. 1 show that the most difficult item (WM1.4) exceeds the person with the highest cognitive capacity. Similarly, there are multiple items located below the person with the lowest capacity.

**Table 2:** Distribution of participants according to their scores

Number of participants achieving the corresponding score N (%)	Total items score N (%)	FaCE score 0-100% (SD)
0 (0)	0 (0)	0 (21.60)
0 (0)	1 (3.70)	13.61 (11.47)
1 (0.41)	2 (7.41)	20.98 (7.91)
0 (0)	3 (11.11)	25.14 (6.39)
2 (0.82)	4 (14.81)	28.06 (5.54)
3 (1.22)	5 (18.52)	30.36 (5.02)
3 (1.22)	6 (22.22)	32.30 (4.68)
11 (4.49)	7 (25.93)	34.03 (4.46)
7 (2.86)	8 (29.63)	35.62 (4.32)
7 (2.86)	9 (33.33)	37.14 (4.24)
14 (5.71)	10 (37.04)	38.62 (4.21)
12 (4.90)	11 (40.74)	40.09 (4.21)
6 (2.45)	12 (44.44)	41.57 (4.24)
24 (9.80)	13 (48.15)	43.07 (4.29)
15 (6.12)	14 (51.85)	44.63 (4.38)
21 (8.57)	15 (55.56)	46.26 (4.49)
12 (4.90)	16 (59.26)	47.98 (4.63)
20 (8.16)	17 (62.96)	49.81 (4.80)
17 (6.94)	18 (66.67)	51.80 (4.99)
12 (4.90)	19 (70.37)	53.94 (5.19)
11 (4.49)	20 (74.07)	56.27 (5.41)
14 (5.71)	21 (77.78)	58.82 (5.69)
17 (6.94)	22 (81.48)	61.67 (6.07)
7 (2.86)	23 (85.19)	65.00 (6.65)
6 (2.45)	24 (88.89)	69.17 (7.59)
2 (0.82)	25 (92.59)	74.92 (9.20)
1 (0.41)	26 (96.30)	84.45 (12.72)
0 (0)	27 (100)	100 (22.46)

Test-of-fit statistics demonstrated outfit residuals between 0.6 and 2.8, meaning that the observed (measured) data fit the predicted model. There are almost no gaps within the scale (less than 0.4 *Logit* differences), as the items are separated by less than 1.5 *Logits* [31]. The polarity of items showed good fit with what was expected by the model as the items fit the hypothesized mathematical model (predictive validity); thus, as the difficulty level of items increases, a higher ability level is required (point measure correlations were positive, ranging from 0.37 to 0.71).

The DIF analysis showed that age, sex, education, cancer stage, chemotherapy and radiotherapy status, comorbidities, and interviewers did not have significant differential functioning (*p* > 0.05).

**Fig. 2:**
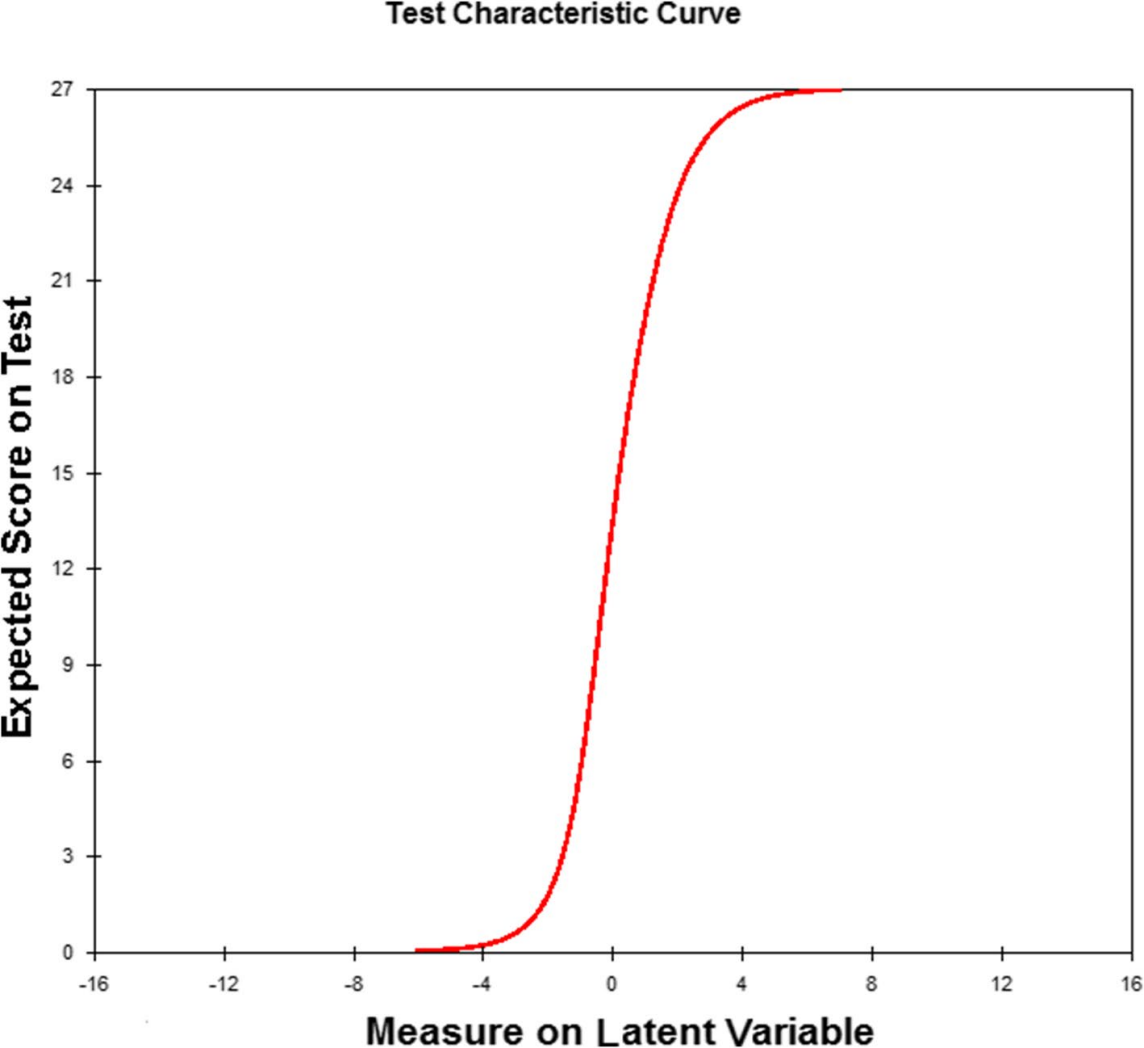
Test Characteristic Curve

The PCA indicated that unidimensionality was satisfied. The explained variance of the items was 52.7% (expected value was 56.2%). No potential sub-dimensions (eigenvalue > 2) were present since no clusters were identified on each contrast (eigenvalues ranged between 0.7 and 1.8). The results of the PCA on the standardized residuals showed no sub-dimension, meaning that FaCE is unidimensional. The item characteristic curves of each item show no major deviation from what was expected by the model. Figure 2 shows the test characteristic curves; the sigmoidal shape illustrates good response probability. FaCE showed excellent item reliability (96%). Item separation indexes confirm that our population size was large enough to test the set of items. Person reliability index of 0.65 and person separation index of 1.37 were observed.

Results of the ANOVA showed no significant effect of the time needed to complete the test on the score (*p* = 0.88) nor of the evaluators on the time needed to complete the test (*p* = 0.85).

### Fast Cognitive Evaluation Scoring System and Interpretation

Table 3 describes FaCE and its scoring system. From left to right, the columns present the assessed cognitive dimensions, the corresponding tasks to be performed by the patient, the response categories for each task, and the attributed values for each response category.

**Table 3:** Description of the Fast Cognitive Evaluation Tool and the Scoring System

**Cognitive dimension**								**Task**									**Response categories**									**Value**					
Working memory								Repeat 7 words (1^st^ try)									0 to 3									0					
																	4									1					
																	5									2					
																	6									3					
																	7									4					
								Repeat 7 words (2^nd^ try)									0 to 4									0					
																	5									1					
																	6									2					
																	7									3					
Visuospatial abilities								Reproduce a cube									Failed									0					
																	Succeeded									1					
Executive functioning								Trail test (A-H; 1-8)									Failed									0					
																	Succeeded									1					
Attention								Perform sequential subtractions									0 or 1									0					
																	2									1					
																	3									2					
																	4									3					
																	5									4					
Verbal fluency								Name as many fruits and vegetables as possible in one minute									0 to 14									0					
																	15 to 16									1					
																	17 to 18									2					
																	19 to 20									3					
																	21 to 22									4					
																	23 to 24									5					
																	25 to 26									6					
																	27 to 29									7					
																	30 or more									8					
Delayed recall								Recall 7 words (given at the beginning of the test)									0 or 1									0					
																	2									1					
																	3									2					
																	4									3					
																	5									4					
																	6									5					
																	7									6					
Total items score																										/27					
**Scale to convert the total items score into the FaCE score**																															
Total items score^a^	0	1	2	3	4	5	6		7	8	9	10	11	12	13	14		15	16	17	18	19	20	21	22		23	24	25	26	27
**FaCE score**^**a**^	**0**	**14**	**21**	**25**	**28**	**30**	**32**		**34**	**36**	**37**	**39**	**40**	**42**	**43**	**45**		**46**	**48**	**50**	**52**	**54**	**56**	**59**	**62**		**65**	**69**	**75**	**84**	**100**

^a^Exact values with decimals and standard deviations are presented in Appendix 4

Individuals earn points for successfully completing items. The total number of points earned is termed the ‘total items score’ (0 to 27 points), which is converted to a percentage ‘FaCE score’ (0 to 100%) using a conversion scale. The conversion scale was created using the *Logit* values obtained by RMT modelling of the total items score converted to a percentage scale. Therefore, the FaCE score is linear, with equidistant scores representing equal differences in cognitive performance, unlike to the total items score, which resembles the MoCA [14]. For instance, if two patients both ‘lose’ three (3) points on their total items score but start with different original scores (e.g., 27 to 24 vs. 25 to 22), they will have experienced different degrees of cognitive decline (30% vs. 12%, respectively). Conversely, if two patients lose the same percentage of their FaCE score, regardless of their original scores (e.g., 90% to 80% vs. 20% to 10%), they have experienced the same degree of cognitive decline (10%). Therefore, the FaCE score behaves like a ruler. In the research context, this means that estimating changes in cognitive performance over time is more accurately done using the linear FaCE score than the non-linear total items score [25].

### Fast Cognitive Evaluation linguistic results

Results from cross-linguistic comparisons showed comparability between the French and English versions of FaCE (appendix 3). Benchmarking of the stimuli and tasks against databases of psycholinguistic norms for both languages offer good face validity that the cognitive demands of both instruments are similar. This methodology is robust and could be used for translation into other languages.

## Discussion

FaCE is an easily administrable tool for clinical and research use with excellent psychometric properties and is suitable for patients living with cancer. It is linear, has no ceiling or floor effects, no gaps along the scale, with good internal consistency and discriminatory power. Only the necessary number of items are used. It accurately evaluates cognitive abilities with minimal interviewer training requirements; thus, it is accessible for any healthcare professional or research staff. A population of cancer patients along the whole spectrum of the illness trajectory, from diagnosis, remission, to advanced stage, were involved, ensuring that FaCE is adapted to all patients with cancer [3].

As previously mentioned, psychometric properties of the MoCA showed a significant floor and ceiling effect [15, 16]. The authors tried to overcome the floor effect of the MoCA by combining it with MMSE [41]. The results showed improved precision in the lower range of patients’ cognitive performance; however, the dimensionality remained questionable, and the ceiling effect could not be overcome. Other authors proposed an extensive cognitive test evaluating five domains for the early recognition of atypical dementia in patients over 50 years old [42, 43]. This test requires 20 to 30 minutes to administer and advanced training. Its psychometric properties have not been tested. Additionally, it may not meet criteria for unidimensionality as the measure includes a behavioral index score reported by caregivers or close relatives. Other domains, such as semantic knowledge, and identification of surface dyslexia or dysgraphia may affect dimensionality. Linearity and floor and ceiling effects have also not been formally tested.

In clinical settings, FaCE could overcome the lack of existing assessment tools that leads to underestimation of cognitive difficulties during cancer treatment and illness trajectory. Subjective complaints of cognitive impairment could be objectively measured using FaCE for better clinical patient-tailored management and improved quality of life [44]. Furthermore, as patients with cancer are often affected by fatigue, sleep disruption, pain, and nutritional deficits, it is important for tools that screen for cognitive changes to be short while being thorough. This measurement tool can track subtle cognitive changes across a wide range of cognitive performance in patients at risk of CRCI even at onset of symptoms.

To overcome any potential learning (practice) effects, two versions of FaCE were developed per language (French and English) based on neuropsychometric and neurolinguistic parameters. Considering the simplicity of the tool, it would be safe to assume that comparison of the different versions would result in similar psychometric properties; this should be confirmed in future research.

### Limitations

Despite approximately half of our sample being breast cancer survivors, this did not influence the results as no significant differences were observed between the scores of participants with a diagnosis of breast cancer and the scores of participants with one of the 10 other cancer types enrolled in this study.

Psychological status, fatigue, sleep disruption, pain, infection, nutritional deficits, and hormonal changes can influence cognitive function [5, 45]. While these aspects were not measured, we can deduce that if they had influenced the score, clusters or extreme scores would have appeared. As the patients with breast cancer were overrepresented, there was concern about the impact of menopausal status on cognitive ability as described in several studies [6], but it was demonstrated that menopausal changes had no effect on CRCI in this population [6].

The design of this study did not allow for the identification of cut-off scores to classify patients according to the degree of cognitive impairment. Further studies testing the behaviour of FaCE in populations without documented illnesses would be necessary for this purpose. However, this tool is sufficiently developed to screen for cognitive changes over time at the patient and population levels. We recommend using FaCE for clinical and research purposes for persons with cancer at any stage of the disease trajectory, including complete remission.

### Future directions

When using the FaCE in clinical or research settings, it would be relevant to explore the effect on cognitive performance of patients’ personal and clinical characteristics, such as family background, history of depression, type and duration of cancer treatments. Given the subjective impact of CRCI on an individual’s functioning [6, 8], it would also be interesting to explore the person’s subjective experience of CRCI by comparing FaCE scores with those of a tool with a subjective component such as FACT-Cog [8]. Furthermore, given that FaCE does not have a multi-tasking item, it would be interesting to explore the addition of such an item. It is also known that an important component of CRCI is the increasing time requirement to perform cognitive tests. It would be interesting to test the value of implementing the time factor into the FaCE score. Since CRCI may be similar to other impairments described by persons with other chronic pathologies than cancer, it would be relevant to explore the psychometric properties of FaCE in these populations.

## Conclusion

In conclusion, the Fast Cognitive Evaluation (FaCE)*,* developed using Rasch Measurement Theory, is a novel, valid, and reliable measurement tool that can be used to accurately screen cognitive deficits in persons with cancer and to track any changes over time. One very important benefit of this screening tool is that it can be administered to nearly any person with cancer by any healthcare provider with minimal training. The detection of cognitive deficits through repeated administration of FaCE should trigger appropriate non-pharmacological and pharmacological interventions for better quality of life of cancer survivors. FaCE could also be considered a reliable and simple measurement tool in research that explores cognitive disorders in people living with cancer.

## Supplementary Information

**Additional file 1.** 

**Additional file 2.** 

**Additional file 3.**

**Additional file 4.**

## Data Availability

The datasets generated during and analyzed during the current study are not publicly available (due to not obtaining participant informed consent for public availability of study data) but are available from the corresponding author on reasonable request.
