# Supplementary material for: The Fast Cognitive Evaluation (FaCE): a screening tool to detect cognitive impairment in patients with cancer
Source: BMC Cancer. 2023 Jan 9;23:35. doi: 10.1186/s12885-022-10470-1 (PMC9830916; doi:10.1186/s12885-022-10470-1)
Supplement: Supplementary file 1 — Additional file 1. [file 12885_2022_10470_MOESM1_ESM.pdf]

Appendix 1: English FaCE Version A

Fast Cognitive Evaluation (FaCE)

ID: ________ Date of Birth: __/__/__ Education:______years Dominant Hand: Left / Right Sex: M / F Date:__/__/__

➢ **The test begins now, start the stopwatch.** — Score

Immediate Memory

Read the words in the table, the subject has to repeat them. The order is not important.
Complete the second attempt, even if the first one is completed perfectly.

	Leg	Wool	Castle	Tulip	Blue	Horse	Potato	Number of recalled words
1st attempt								____/7

0-3 : 0 points, 4 : 1 point, 5 : 2 points, 6 : 3 points, 7 : 4 points — /4

	Leg	Wool	Castle	Tulip	Blue	Horse	Potato	Number of recalled words
2nd attempt								____/7

0-4 : 0 points, 5 : 1 point, 6 : 2 points, 7 : 3 points — /3

Executive/Visuospatial Functions

Complete the alphanumeric trajectory — Task succeded : Yes ❑ No ❑

Success : 1 point

Draw a cube — Task succeded : Yes ❑ No ❑

Success : 1 point

/2

Attention

Substract 7 from 101 and continue to substract 7 from the result, even if the result is wrong.

94☐ 87☐ 80☐ 73☐ 66☐ Other results ____________________________

Number of successive correct substractions: ______/5

0-1 : 0 points, 2 : 1 point, 3 : 2 points, 4 : 3 points, 5 : 4 points — /4

Verbal fluidity

Name as much fruits and vegetables as you can in a minute. Write down the words named on the second page.

Number of named fruits and vegetables: _____________

0-14 : 0 points, 15-16 : 1 point, 17-18 : 2 points, 19-20 : 3 points, 21-22 : 4 points, 23-24 : 5 points, 25-26 : 6 points, 27-28-29 : 7 points, 30 + : 8 points — /8

Delayed Recall

Number of recalled words : ________/7

	Leg	Wool	Castle	Tulip	Blue	Horse	Potato
7 words recall without help nor hints							

0-1 : 0 points, 2 : 1 point, 3 : 2 points, 4 : 3 points, 5 : 4 points, 6 : 5 points, 7 : 6 points — /6

➢ **The tests ends here. Stop the stopwatch.**

Test Duration: ______min______sec Administered by: ___________

Total /27

Score	%
27	100
26	84
25	75
24	69
23	65
22	62
21	59
20	56
19	54
18	52
17	50
16	48
15	46
14	45
13	43
12	42
11	40
10	39
9	37
8	36
7	34
6	32
5	30
4	28
3	25
2	21
1	14
0	0

©GagnonBruno2016

Two versions in French and in two English are available from corresponding author: Gagnon.Bruno@crchudequebec.ulaval.ca

Complete the alphanumeric trajectory

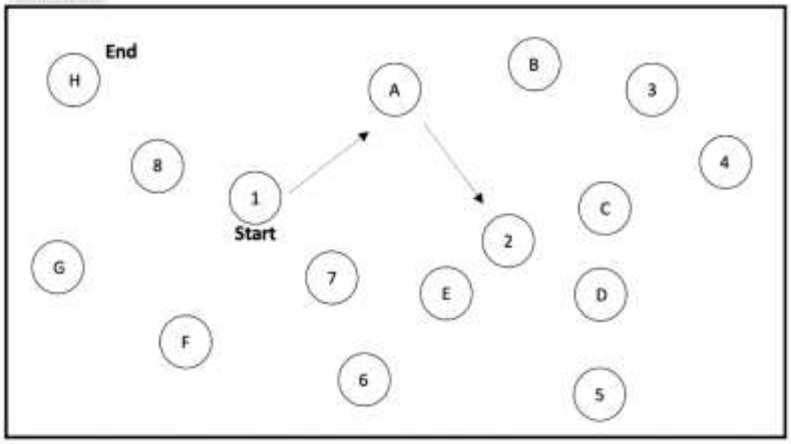

Draw a cube

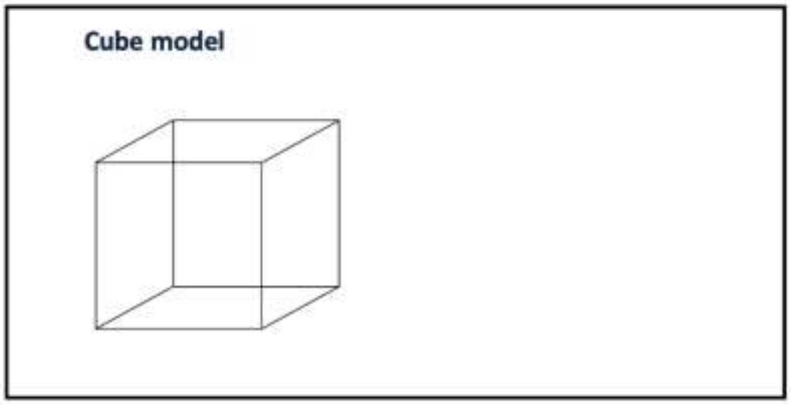

Verbal fluidity Name as much fruits and vegetables as you can in a minute.

_______________ _______________ _______________ _______________ _______________

_______________ _______________ _______________ _______________ _______________

_______________ _______________ _______________ _______________ _______________

_______________ _______________ _______________ _______________ _______________

_______________ _______________ _______________ _______________ _______________

_______________ _______________ _______________ _______________ _______________

_______________ _______________ _______________ _______________ _______________

_______________ _______________ _______________ _______________ _______________

©GagnonBruno2016

Two versions in French and in two English are available from corresponding author: Gagnon.Bruno@crchudequebec.ulaval.ca

Appendix 1: English FaCE Version B

Fast Cognitive Evaluation (FaCE)

ID: ________ Date of Birth: __/__/__ Education:_______years Dominant Hand: Left / Right Sex: M / F Date:__/__/__

➢ **The test begins now, start the stopwatch.**

Immediate Memory

Read the words in the table, the subject has to repeat them. The order is not important.
Complete the second attempt, even if the first one is completed perfectly.

	Neck	Wax	Cable	Turnip	Red	Fish	Violin	Number of recalled words
1st attempt								____/7

0-3 : 0 points, 4 : 1 point, 5 : 2 points, 6 : 3 points, 7 : 4 points

Score: /4

	Neck	Wax	Cable	Turnip	Red	Fish	Violin	Number of recalled words
2nd attempt								____/7

0-4 : 0 points, 5 : 1 point, 6 : 2 points, 7 : 3 points

Score: /3

Executive/Visuospatial Functions

Complete the alphanumeric trajectory Task succeded : Yes ❑ No ❑

Success : 1 point

Draw a pyramid Task succeded : Yes ❑ No ❑

Success : 1 point

Score: /2

Attention

Substract 7 from 103 and continue to substract 7 from the result, even if the result is wrong.

96□ 89□ 82□ 75□ 68□ Other results ____________________________

Number of successive correct substractions: ______/5

0-1 : 0 points, 2 : 1 point, 3 : 2 points, 4 : 3 points, 5 : 4 points

Score: /4

Verbal fluidity

Name as much animals as you can in a minute. Write down the words named on the second page.

Number of named animals : ____________

0-14 : 0 points, 15-16 : 1 point, 17-18 : 2 points, 19-20 : 3 points, 21-22 : 4 points, 23-24 : 5 points, 25-26 : 6 points, 27-28-29 : 7 points, 30 + : 8 points

Score: /8

Delayed Recall

Number of recalled words : ________/7

	Neck	Wax	Cable	Turnip	Red	Fish	Violin
7 words recall without help nor hints							

0-1 : 0 points, 2 : 1 point, 3 : 2 points, 4 : 3 points, 5 : 4 points, 6 : 5 points, 7 : 6 points

Score: /6

➢ **The test ends here. Stop the stopwatch.**

Test Duration: ______min______sec Administered by: ___________

Total /27

Score	%
27	100
26	84
25	75
24	69
23	65
22	62
21	59
20	56
19	54
18	52
17	50
16	48
15	46
14	45
13	43
12	42
11	40
10	39
9	37
8	36
7	34
6	32
5	30
4	28
3	25
2	21
1	14
0	0

©GagnonBruno2016

Complete the alphanumeric trajectory

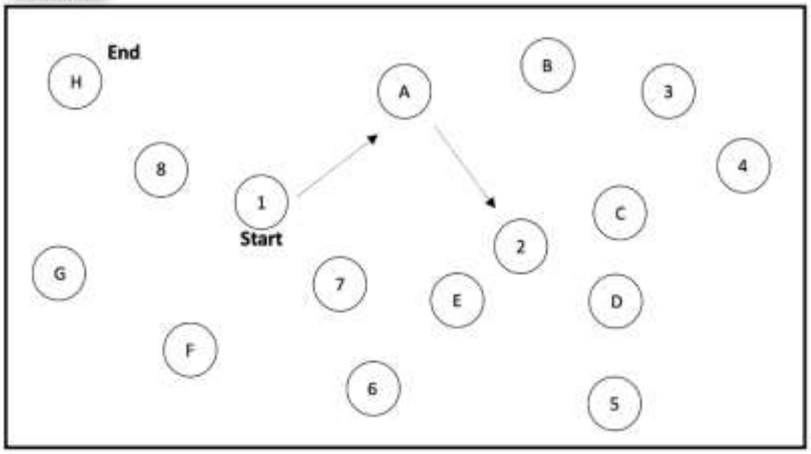

Draw a cube

Cube model

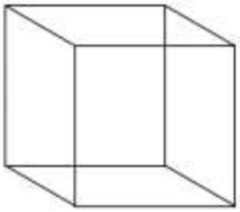

Verbal fluidity Name as much animals as you can in a minute.

©GagnonBruno2016

Appendix 1: Training Version

Fast Cognitive Evaluation (FaCE)
(Training)

ID: ________ Date of Birth: __/__/__ Education:_______years Dominant Hand: Left / Right Sex: M / F Date:__/__/___

Orientation : Date ☐ Month ☐ Year ☐ Day ☐ Season ☐ City ☐<br>➢ **The test begins now, start the stopwatch.**	Score	%
**Immediate Memory**<br>Read the words in the table, the subject has to repeat them. The order is not important.<br>Complete the second attempt, even if the first one is completed perfectly.		
**Executive/Visuospatial Functions**<br>Complete the alphanumeric trajectory Task succeded : Yes ❑ No ❑ | Draw the shape Task succeded : Yes ❑ No ❑		
**Attention**<br>Substract 2 from 12 and continue to substract 2 from the result, even if the result is wrong.<br>10☐ 8☐ 6☐ 4☐ 2☐ Other results ___________________________		
**Verbal fluidity** Name as much items you can find on a dinner table in 15 seconds. Write down the words named on the second page.<br>Number of items: ____________		
**Delayed Recall** Number of recalled words: ________/3		
➢ **The test ends here. Stop the stopwatch.**<br>Test Duration: _____min_____sec Administered by: ___________ — **Total**		

Immediate Memory — 1st attempt:

	Arm	Cotton	House	Number of recalled words
1st attempt				____/3

Immediate Memory — 2nd attempt:

	Arm	Cotton	House	Number of recalled words
2nd attempt				____/3

Delayed Recall:

	Arm	Cotton	House
3 words recall without help nor hints			

©GagnonBruno2016

Complete this alphanumeric trajectory

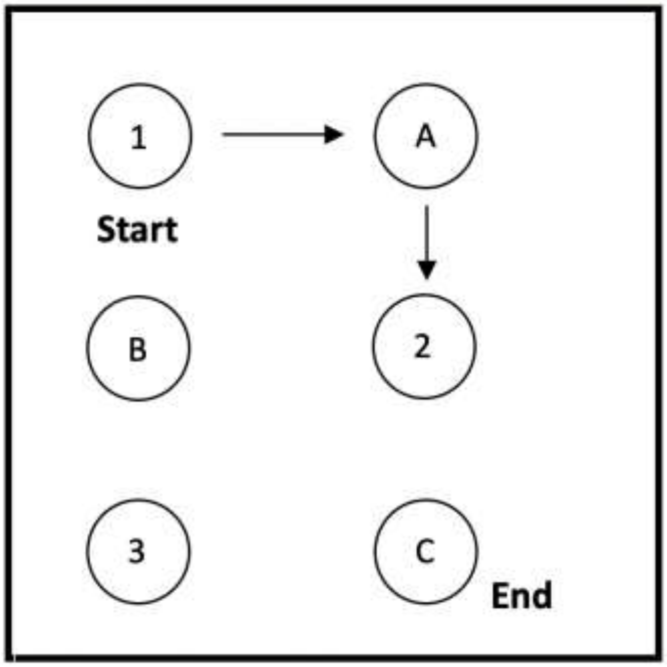

Draw this shape

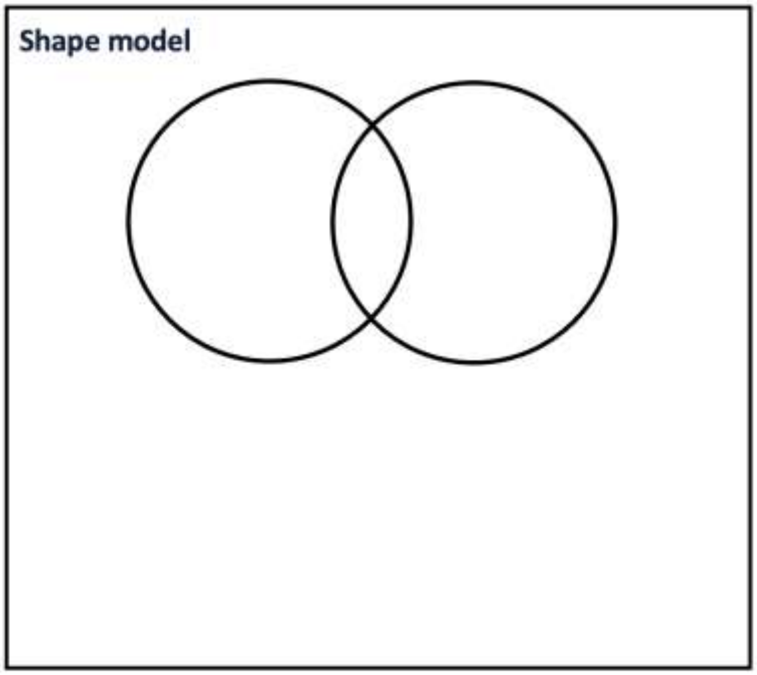

Verbal fluidity Name as much items you can find on a dinner table during a family dinner in 15 seconds.

__________________ __________________ __________________ __________________ __________________

__________________ __________________ __________________ __________________ __________________

©GagnonBruno2016

Fast Cognitive Evaluation
FaCE

Instructions for Administration

The FaCE questionnaire is an instrument conceived to quickly assess cancer patients' cognitive functions and follow their evolution. It assesses attention, memory, visuocontruction and executive functions. It is printed on both sides of a page. It is scored from 0 to 100 and is usually completed in 6 minutes.

Required Material:

Pencils (different types and colors)
Stopwatch: two or one stopwatches with the possibility to do two simultaneous timings
Testing sheet (printed on both sides)
Calm room

1- General Information:

Before beginning the test, complete the general information in the document's first section.

- **ID**: The individual's identification number

Write down the individual's name and/or the file number if it is a clinical follow-up, or his identification number as assigned if it is a research project.

- **Education**: number of completed years.

Ask: *"Until now, how many school years have you completed?"*
If the person struggles with counting the number of school years he/she has completed, add them up by asking the highest education level reached and help him/her calculate the total based on this data: completed elementary school: 6 years; completed high school : 5 years; CEGEP and university depending on the program studied.
Write down the number of school years completed.

- **Date of Birth**

Ask for the individual's date of birth and enter it in the day/month/year format (DD/MM/YYYY)
Ask: *"What is your date of birth?"*

- **Dominant Hand**

Document the individual's dominant hand. Ask: "*Which hand is your dominant one?"*
Circle Left if he's left-handed, and Right, if he's right-handed.

- **Sex**

Ask the person to indicate their sex by saying *"What is your sex?"* and circle the letter corresponding to his sex

F, if she's a female,
M, if he's a male,
Other: for any other answer (does not want to answer, does not know, etc.)

- **Date**

Write down the date during which the test is administered (day/month/year: DD/MM/YYYY).

©Bruno Gagnon2016

2- **Orientation**:

While not being part of the test itself, this section will facilitate its beginning.
Say *"We will start by a practice question that will not contribute to your test's final score. What is the date today?"*
If the given answer is incomplete, ask for precisions: *"Tell me today's year, month, date and the exact day"*.
Then, ask: *"What season are we currently in?"*
Finally, ask information on the location: *"Tell me the name of the city we currently are in?"*
For each of the elements (date, month, year, day, season, city) check the corresponding box if the answer is correct.

Beginning of the Test

Inform the person that the test will now begin and that you will start the stopwatch.
"The test will now begin. I will ask you questions to which you will try to answer to the best of your ability. Pay attention. If anything escapes you, do not hesitate to ask me questions. I will now start the stopwatch."

Start the first stopwatch.

- ***Before beginning the next part, the first stopwatch has to be started because the test now begins.***

3- **Immediate Memory:**

Inform the individual that 7 words will be enunciated and that he has to pay attention because he will have to repeat them once they are all enunciated. Make sure that he clearly understands the assignment.

- **1st Try**

Read the 7-word list, clearly and audibly, at the rate of one word per second. If the individual repeats the correct word, check the box that corresponds to this word.
To inform the subject, say: *"I will read a wordlist that you will have to remember. Pay close attention. When I will be done, I want you to tell me as many words as possible that you remember from that list, in any given order"*.

- **2nd Try**

When the subject has completed his first try, no matter how many words were recalled, reread the 7-word list at the same rate of one word per second. After giving out the necessary instructions and checking the box corresponding to the words that the subject recalled during his first try, tell him: *"Now, I will read the same wordlist a second time. Try to remember and tell me as many words as you can from that list, even the ones that you recalled during your first try, in any given order"*.
When the second try is done, inform the subject that he will have to remember these words because he will have to tell them again at the very end of the test. "*Try to remember the words from the list, I will ask you to repeat them to me at the end of the test.*"

4- **Executive and Visuospatial Functions**:

Show the person different kinds of pencils, so that they can choose the one that suits him best. Flip the document and show him the two sections that are on the other side.

- **1st Section: Alphanumeric Trajectory**

Inform the individual that he has to connect the letters and numbers on the page.
Tell the instructions while pointing the appropriate spots on the document: *"I want you to draw a line without lifting the pencil, alternating from a number to a letter, while respecting the ascending numerical order and the alphabetical order. Start here (indicate 1) and draw the line to the letter A, then to the 2, and so on. End here" (indicate the H).*

©Bruno Gagnon2016

- **2nd Section: 3D Cube Drawing**

Ask the subject to reproduce the presented cube. Say: *"I want you to copy this drawing in the most accurate way possible"*, while pointing the cube.

5- Attention

Ask the subject to substract "7" from '101' and to continue to substract "7" from the result of the first substraction, until he is asked to stop. Make sure that he clearly understands the assignment.
Give out the following instructions: *"Now I want you to calculate 101 - 7, and then, continue to substract 7 from your answer, until I ask you to stop"*. If necessary, you can repeat the instructions a second time.
Once the individual has completed 5 substractions, ask him to stop.
If the FaCE is to be administered during a future visit, perform the previous steps starting the subtractions with '103' instead of '101'.
Check the appropriate box for each result of the subtractions performed. If the individual fails the first subtraction or a subsequent subtraction, do not interrupt him and write down in the "Other Results" section the result obtained. The individual should continue to subtract "7" from the result even if it is wrong, and so on. Then, validate the number of successful subtractions.

6- Language and Speed of Thought

Inform the individual that when you will give him a signal, he will have to name as much fruits and vegetables as possible in a minute, in any given order. Make sure that he clearly understands the assignment: *"I want you to name me as much different fruits and vegetables as possible. Do not name varieties of the same fruit or vegetable, for example, green grapes and red grapes. I will ask you to stop when a minute has passed. Are you ready? You can now start"*.
Give the individual the signal to start and start the second stopwatch.
Write down the names of the fruits and vegetables enunciated by the individual or simply write down a check mark for every word named. If the same word is repeated, it will only be counted once. If varieties of the same fruit or vegetable are named (example: red grape, green grape), this will only count for a single fruit or a single vegetable.
When a minute has passed, inform the individual that the assignment is complete and stop the second stopwatch.

7- Delayed Recall

Ask the subject to name the words from the 7-word list enunciated at the beginning of the test.
"Earlier, I read out loud a wordlist that I asked you to remember. Now, I want you to name every word that you remember from that list."

End of Test

- ***The test ends here, stop the first stopwatch.***

Write down the duration of the test in minutes in the section "Test Duration".
Write down the professional who administered the FaCE's initials in the section 'Administered by'.

©Bruno Gagnon2016

Test Scoring

To calculate the individual's test final score, add up every point obtained in each task. This score corresponds to the individual's total and final score to the test. To find out the corresponding percentage to the individual's final score, please refer to the table on the right of the first page of the test. For a more precise corresponding percentage, please refer to the table below.

Score	Percentage (%)	Score	Percentage (%)
0	0,00	16	47,98
1	13,61	17	49,81
2	20,98	18	51,80
3	25,14	19	53,94
4	28,06	20	56,27
5	30,36	21	58,82
6	32,30	22	61,67
7	34,03	23	65,00
8	35,62	24	69,17
9	37,14	25	74,92
10	38,62	26	84,45
11	40,09	27	100,00
12	41,57		
13	43,07		
14	44,63		
15	46,26		

©Bruno Gagnon2016

Appendix 1: French FaCE Version A

Fast Cognitive Evaluation (FaCE)

ID: ________ Date de naissance: __/__/__ Éducation:__________ans Main dominante : Gauche / Droite Sexe : M / F Date:__/__/___

➢ **Le test débute ici, enclencher le chronomètre.** — Score

Mémoire immédiate

Lisez les mots dans le tableau, le participant doit les répéter. L'ordre n'a pas d'importance.
Effectuer le deuxième essai, même si le premier est réussi.

	Jambe	Laine	Château	Tulipe	Bleu	Cheval	Patate	Nombre de mots répétés
1er essai								____/7

0-3 : 0 points, 4 : 1 point, 5 : 2 points, 6 : 3 points, 7 : 4 points — /4

	Jambe	Laine	Château	Tulipe	Bleu	Cheval	Patate	Nombre de mots répétés
2e essai								____/7

0-4 : 0 points, 5 : 1 point, 6 : 2 points, 7 : 3 points — /3

Fonctions exécutives/visuo-spatiales

Faire un tracé alphanumérique Tâche réussie : Oui ❑ Non ❑

Réussite : 1 point

Dessiner un cube Tâche réussie : Oui ❑ Non ❑

Réussite : 1 point

/2

Attention

Soustraire 7 de 101 et continuer à soustraire 7 du résultat, même si le résultat est erroné.

94☐ 87☐ 80☐ 73☐ 66☐ Autres résultats ____________________________

Nombre de soustractions correctes consécutives : ______/5

0-1 : 0 points, 2 : 1 point, 3 : 2 points, 4 : 3 points, 5 : 4 points — /4

Langage et rapidité d'idéation

Nommer le maximum de fruits et légumes en 1 minute. Écrire les mots énoncés au verso.

Nombre de fruits et légumes nommés : _____________

0-14 : 0 points, 15-16 : 1 point, 17-18 : 2 points, 19-20 : 3 points, 21-22 : 4 points, 23-24 : 5 points, 25-26 : 6 points, 27-28-29 : 7 points, 30 + : 8 points — /8

Rappel différé

Nombre de mots rappelés : ________/7

	Jambe	Laine	Château	Tulipe	Bleu	Cheval	Patate
Rappel des 7 mots sans aide ni indice							

0-1 : 0 points, 2 : 1 point, 3 : 2 points, 4 : 3 points, 5 : 4 points, 6 : 5 points, 7 : 6 points — /6

➢ **Le test se termine ici. Arrêter le chronomètre.**

Durée du test : _____min_____sec Administré par : ___________

Total — /27

Score	%
27	100
26	84
25	75
24	69
23	65
22	62
21	59
20	56
19	54
18	52
17	50
16	48
15	46
14	45
13	43
12	42
11	40
10	39
9	37
8	36
7	34
6	32
5	30
4	28
3	25
2	21
1	14
0	0

©GagnonBruno2016

Faire un tracé alphanumérique

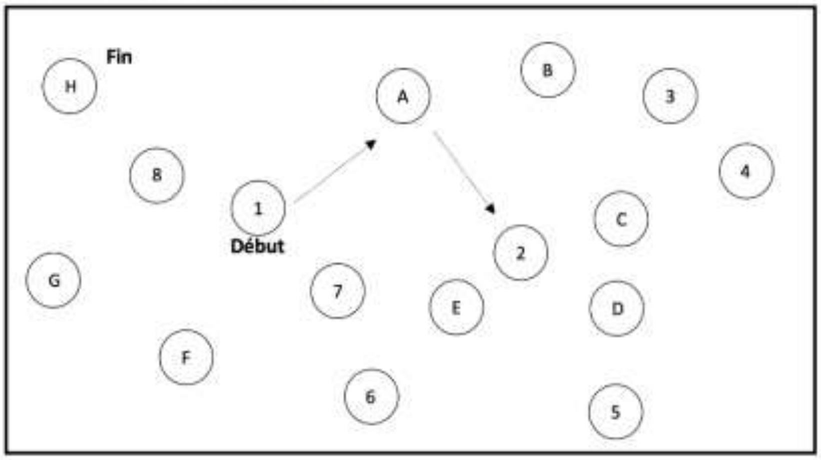

Dessiner un cube

Modèle de cube

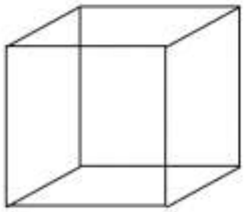

Langage et rapidité d'idéation Nommer le maximum de fruits et légumes en 1 minute.

©GagnonBruno2016

Appendix 1: French FaCE Version B

Fast Cognitive Evaluation (FaCE)

ID: ________ Date de naissance: __/__/__ Éducation:__________ans Main dominante : Gauche / Droite Sexe : M / F Date:__/__/___

➢ **Le test débute ici, enclencher le chronomètre.** — Score

Mémoire immédiate

Lisez les mots dans le tableau, le participant doit les répéter. L'ordre n'a pas d'importance.
Effectuer le deuxième essai, même si le premier est réussi.

	Cou	Cire	Câble	Navet	Rouge	Poisson	Violon	Nombre de mots répétés
1er essai								____/7

0-3 : 0 points, 4 : 1 point, 5 : 2 points, 6 : 3 points, 7 : 4 points — Score : /4

	Cou	Cire	Câble	Navet	Rouge	Poisson	Violon	Nombre de mots répétés
2e essai								____/7

0-4 : 0 points, 5 : 1 point, 6 : 2 points, 7 : 3 points — Score : /3

Fonctions exécutives/visuo-spatiales

Faire un tracé alphanumérique Tâche réussie : Oui ☐ Non ☐

Réussite : 1 point

Dessiner une pyramide Tâche réussie : Oui ☐ Non ☐

Réussite : 1 point

Score : /2

Attention

Soustraire 7 de 103 et continuer à soustraire 7 du résultat, même si le résultat est erroné.

96☐ 89☐ 82☐ 75☐ 68☐ Autres résultats ____________________________

Nombre de soustractions correctes consécutives : ______/5

0-1 : 0 points, 2 : 1 point, 3 : 2 points, 4 : 3 points, 5 : 4 points — Score : /4

Langage et rapidité d'idéation

Nommer le maximum d'animaux en 1 minute. Écrire les mots énoncés au verso.

Nombre d'animaux nommés : ____________

0-14 : 0 points, 15-16 : 1 point, 17-18 : 2 points, 19-20 : 3 points, 21-22 : 4 points, 23-24 : 5 points, 25-26 : 6 points, 27-28-29 : 7 points, 30 + : 8 points — Score : /8

Rappel différé

Nombre de mots rappelés : ________/7

	Cou	Cire	Câble	Navet	Rouge	Poisson	Violon
Rappel des 7 mots sans aide ni indice							

0-1 : 0 points, 2 : 1 point, 3 : 2 points, 4 : 3 points, 5 : 4 points, 6 : 5 points, 7 : 6 points — Score : /6

➢ **Le test se termine ici. Arrêter le chronomètre.**

Durée du test : _____min_____sec Administré par : __________

Total /27

Score	%
27	100
26	84
25	75
24	69
23	65
22	62
21	59
20	56
19	54
18	52
17	50
16	48
15	46
14	45
13	43
12	42
11	40
10	39
9	37
8	36
7	34
6	32
5	30
4	28
3	25
2	21
1	14
0	0

©GagnonBruno2016

Faire un tracé alphanumérique

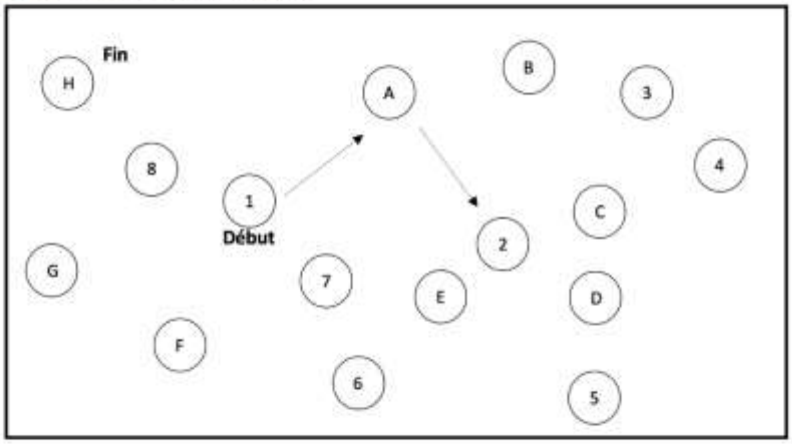

Dessiner un cube

Modèle de cube

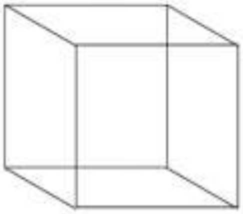

Langage et rapidité d'idéation Nommer le maximum d'animaux en 1 minute.

_______________ _______________ _______________ _______________ _______________

_______________ _______________ _______________ _______________ _______________

_______________ _______________ _______________ _______________ _______________

_______________ _______________ _______________ _______________ _______________

_______________ _______________ _______________ _______________ _______________

_______________ _______________ _______________ _______________ _______________

_______________ _______________ _______________ _______________ _______________

_______________ _______________ _______________ _______________ _______________

©GagnonBruno2016

Fast Cognitive Evaluation
FaCE

Instructions pour l'administration

Le questionnaire FaCE est un instrument conçu pour faire une évaluation rapide de la fonction cognitive des patients avec cancer et afin de suivre leur évolution. Il évalue l'attention, la mémoire, les fonctions visuo-constructives et exécutives. Il est imprimé sur une feuille en recto-verso. Il est coté de 0 à 100 et se complète en moyenne en 6 minutes.

Matériel nécessaire :

Crayons (différents types et couleurs)
Chronomètre : deux ou un chronomètre avec possibilité de faire deux chronométrages simultanés
Feuille de test (recto-verso)
Salle calme

1- Informations générales :

Avant de commencer le test, complétez les informations générales de la 1ère section du document.

- **ID** : numéro d'identification de l'individu

Inscrivez le nom et/ou le numéro de dossier de la personne s'il s'agit d'un suivi clinique, ou son numéro d'identification tel qu'il a été attribué s'il s'agit d'un projet de recherche.

- **Éducation** : nombre d'années d'études complétées.

Dites : *«Quel est le nombre total d'années d'études que vous avez complétées jusqu'ici.»*
Si la personne a de la difficulté à calculer le nombre d'années d'études, demandez-lui son plus haut niveau de scolarité et aidez-la à faire les calculs en se basant sur les données suivantes : primaire complété : 6 années; secondaire complété : 5 années; CEGEP et université selon le programme.
Inscrivez le nombre total d'années d'études complétées.

- **Date de naissance**

Demandez la date de naissance de l'individu et inscrivez-la selon le format jour/mois/année (JJ/MM/AAAA)
Dites : *«Quelle est votre date de naissance?»*

- **Main dominante**

Documentez la main dominante de l'individu. Dites : *«Quelle est votre main dominante ?»*
Entourez Gauche, s'il est gaucher, et Droite, s'il est droitier.

- **Sexe**

Demandez à la personne d'indiquer son sexe en disant *«Quel est votre sexe? »* et entourez la lettre correspondant à son sexe

F, si c'est une femme,
M, si c'est un homme
Autre : pour toute autre réponse (ne veut pas répondre, ne sait pas, etc.)

- **Date**

Inscrivez la date à laquelle le test est administré (jour/mois/année : JJ/MM/AAAA).

©Bruno Gagnon2016

2- **Orientation** :

Cette section servira à faciliter le début du test mais n'en fait pas partie proprement dit.
Dites *«Nous allons commencer par une question d'entraînement qui ne sera pas comptée dans le test. Quelle est la date d'aujourd'hui ?»*
Si la réponse fournie est incomplète, demandez des précisions : *«Dites-moi l'année, le mois, la date du jour, et le jour exact».*
Ensuite, demandez : *«En quelle saison sommes-nous actuellement?»*
Pour terminer, demandez des informations sur le lieu : *«Dites-moi le nom de la ville où nous sommes présentement?»*
Pour chacun des éléments (date, mois, année, jour, saison, ville) cochez la case correspondante si la réponse est exacte.

Début du test

Informez la personne que le test va maintenant commencer et que vous allez démarrer le chronomètre.
«Le test va maintenant commencer. Je vais vous poser les questions auxquelles vous tenterez de répondre de votre mieux. Soyez attentif. Si des éléments vous échappent, n'hésitez pas à me poser des questions. Je vais maintenant démarrer le chronomètre.»

Démarrez le premier chronomètre.

- ***Avant de commencer la partie suivante, le premier chronomètre doit être enclenché, car le test débute à ce moment.***

3- **Mémoire immédiate :**

Informez l'individu que 7 mots vont lui être nommés et qu'il doit y prêter attention, car il va devoir les répéter à la fin de leur énoncé. Assurez-vous qu'il a bien compris l'exercice.

- **1er essai**

Lisez la liste de 7 mots, au rythme de un mot par seconde, de façon claire et audible. Si le mot est répété par l'individu, faites un crochet à l'espace correspondant.
Pour informer le sujet, dites : *«Je vais vous lire une liste de mots que vous aurez à retenir. Écoutez-les attentivement. Quand j'aurai terminé, je veux que vous me redisiez le plus de mots possible dont vous vous rappelez, dans l'ordre que vous voulez».*

- **2ème essai**

Lorsque le sujet a terminé son premier essai, peu importe le nombre de mots dont il s'est rappelé, relisez la liste de 7 mots, toujours au rythme d'un mot par seconde. Après avoir donné les instructions nécessaires et marqué d'un crochet ceux qu'il aura répété lors de ce second essai, dites-lui : *«Maintenant je vais lire la même liste de mots une seconde fois. Essayez de vous rappeler du plus grand nombre de mots possible, y compris ceux que vous avez énoncés la première fois et dites les moi, dans l'ordre que vous souhaitez».*
À la fin du deuxième essai, informez le sujet qu'il devra retenir ces mots, car il aura à les redire à la fin du test. «*Gardez-en mémoire les mots que je vous ai lus, je vais vous demander de les répéter à la fin du test.*»

4- **Fonctions exécutives et visuo-spatiales** :

Présentez à la personne des crayons de différentes sortes, afin qu'il choisisse celui qui lui convient le mieux. Retournez le document, au verso il y a deux sections, présentez-le lui alors.

- **1ère section: Tracé alphanumérique**

Informez l'individu qu'il doit relier les lettres et les chiffres présentés.
Dire les instructions en indiquant les endroits appropriés : *« Je veux que vous traciez une ligne sans lever le crayon, en alternant d'un chiffre à une lettre, tout en respectant l'ordre numérique ascendant et l'ordre alphabétique. Commencez ici* (indiquez le 1) *et tracez la ligne vers la lettre A, ensuite vers le 2, et ainsi de suite. Terminez ici »* (indiquez le H).

- **2ème section : Dessin du cube**

Demandez à la personne de reproduire le cube. Dites : *«Je veux que vous copiez ce dessin le plus précisément possible»*, en indiquant le cube.

5- Attention

Demandez à l'individu de soustraire 7 du nombre '101' et de continuer à soustraire « 7 » du résultat obtenu de la première soustraction jusqu'à ce qu'il lui soit demandé de s'arrêter. Assurez-vous que l'exercice demandé soit bien compris.
Donnez les instructions suivantes : *«Maintenant je veux que vous calculiez 101 - 7, et ensuite, continuez de soustraire 7 de votre réponse, jusqu'à ce que je vous dise d'arrêter»*. Vous pouvez répéter les instructions une deuxième fois si nécessaire.
Une fois que l'individu a effectué 5 soustractions, demandez-lui de s'arrêter.
Si le FaCE est administré lors d'une prochaine visite, effectuez les étapes précédentes en commençant les soustractions par '103 '.
Cochez la case appropriée pour chaque résultat des soustractions effectuées. Si l'individu échoue à la première soustraction ou à une soustraction ultérieure, ne l'interrompez pas et notez dans la partie « Autres résultats » le résultat obtenu. L'individu devra continuer à soustraire « 7 » du résultat obtenu même s'il est erroné et ainsi de suite. Validez ensuite le nombre de soustractions réussies.

6- Langage et rapidité d'idéation

Informez l'individu qu'au signal que vous lui donnerez, il devra nommer le maximum possible de fruits et de légumes, peu importe leur ordre, en l'espace d'une minute. Assurez-vous que l'exercice est bien compris: *«Je veux que vous me disiez le plus de noms de fruits et légumes différents possible. Ne dites pas plusieurs variétés du même fruit ou légume, par exemple, raisins verts et raisins rouges. Je vais vous dire d'arrêter après une minute. Êtes-vous prêt ? Maintenant, commencez»*.
Donnez le signal de début à l'individu et enclenchez le second chronomètre.
Inscrivez les noms de fruits et de légumes nommés ou simplement inscrire une coche pour chaque mot nommé. Si le même mot est répété, celui-ci ne sera compté qu'une seule fois. Si plusieurs variétés du même fruit ou légume sont nommés (exemple : raisin rouge, raisin vert), cela comptera pour un seul fruit ou un seul légume.
Au bout d'une minute, informez l'individu que l'exercice est terminé et arrêtez le second chronomètre.

7- Rappel différé

Demandez à la personne de nommer les 7 mots énoncés au début du test.
«Je vous ai lu une série de mots plus tôt dont je vous ai demandé de vous rappeler. Maintenant, dites-moi tous les mots dont vous vous rappelez».

Fin du test

- ***Le test se termine ici, arrêtez le premier chronomètre.***

Inscrivez la durée du test en minutes dans la partie appropriée « Durée du test ».
Inscrivez les initiales de l'intervenant qui a administré le FaCE dans la partie appropriée 'Administré par'.

©Bruno Gagnon2016

Cotation du test

Pour coter le test, additionner les points accumulés à chaque épreuve. Le résultat correspond au score total de l'individu au test. Pour connaître le pourcentage correspondant au score total de l'individu, veuillez vous référer au tableau se trouvant à droite de la première page du test. Pour obtenir un pourcentage correspondant plus précis, veuillez vous référer au tableau ci-dessous.

Score	Pourcentage (%)	Score	Pourcentage (%)
0	0,00	16	47,98
1	13,61	17	49,81
2	20,98	18	51,80
3	25,14	19	53,94
4	28,06	20	56,27
5	30,36	21	58,82
6	32,30	22	61,67
7	34,03	23	65,00
8	35,62	24	69,17
9	37,14	25	74,92
10	38,62	26	84,45
11	40,09	27	100,00
12	41,57		
13	43,07		
14	44,63		
15	46,26		

©Bruno Gagnon2016
