# Supplementary material for: The Fast Cognitive Evaluation (FaCE): a screening tool to detect cognitive impairment in patients with cancer
Source: BMC Cancer. 2023 Jan 9;23:35. doi: 10.1186/s12885-022-10470-1 (PMC9830916; doi:10.1186/s12885-022-10470-1)
Supplement: Supplementary file 2 — Additional file 2. [file 12885_2022_10470_MOESM2_ESM.pdf]

Appendix 2 : Fast Cognitive Screen (FCS)

Questionnaire cognitif bref

Fast Cognitive Screen (FCS)

ID :
Scolarité : Date de naissance :
Sexe : Date :

	Points
**Orientation**<br>Date □ Mois □ Année □ jour □ Saison □ Ville □	____/6
**Mémoire**<br>Lire la liste de mots et le participant doit répéter. Faire les deux essais même si le 1er est réussi.	____/7<br>____/0

	jambe	laine	château	tulipe	bleu	cheval	patate
1er essai							
2e essai							

Visuospatial/Éxécutif

Section 1

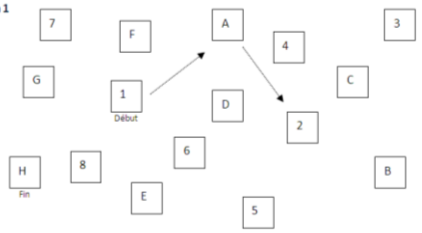

Section 2 Copier le cube

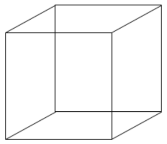

3D=1pt, Parfaitement reproduit=2pt

Section 3 Dessiner une horloge qui indique 14h40.

____/6

Attention

Lire la série de chiffres (1 chiffre/sec.)

Le patient doit la répéter.
5971 □
Le patient doit la répéter à l'envers.
581 □
6849 □
24368 □
142540 □
9781426 □

____/6

Soustraire série de 7 à partir de 101.

94□ 87□ 80□ 73□ 66□

____/5

Langage

Nommer un maximum de mots de la catégorie **Fruits et Légumes** en 1 min (minimum de 20 fruits et/ou légumes pour obtenir le point).
Inscrire les mots nommés par le participant :

____/1

Rappel

____/7

Doit se souvenir des mots sans indices	Jambe □	Laine □	Château □	Tulipe □	Bleu □	Cheval □	Patate □

Temps requis pour le test : __________min
Ajout d'un point si scolarité < 12 ans ____/1

Total : _______/38

Administré par :_______________________________________
