# Supplementary material for: The Fast Cognitive Evaluation (FaCE): a screening tool to detect cognitive impairment in patients with cancer
Source: BMC Cancer. 2023 Jan 9;23:35. doi: 10.1186/s12885-022-10470-1 (PMC9830916; doi:10.1186/s12885-022-10470-1)
Supplement: Supplementary file 3 — Additional file 3. [file 12885_2022_10470_MOESM3_ESM.pdf]

Appendix 3: Additional notes on the linguistic components of FaCE.

These notes provide more details on the development of the English and French versions of the FaCE, with respect to their psycholinguistic properties. To ensure similarity for the 'Immediate Memory'/'Delayed Recall' subtests in the French and English versions of FaCE, English and French stimuli were compared across a set of psycholinguistic variables known to affect verbal working memory recall and lexical access. These included word frequency, word/syllable length, imageability, age of acquisition, and reaction time norms in lexical decision tasks. Put simply, words that are higher frequency, acquired earlier in language acquisition, and are easier to create mental images of, have shorter reaction times and are easier to recall from semantic memory. Further, the number of syllables per word inversely correlates with the number of stimuli that can be recalled in a working memory span task. Thus, significant mismatches across these parameters could lead to differential cognitive demands cross-linguistically on the English and French versions of FacE. For French stimuli, imageability and age of acquisition ratings were derived from Ferrand et al. (2008) and Desrochers and Thompson (2009), while syllable lengths, normed frequency and reaction time norms were drawn from Ferrand et al. (2010). For English translation equivalents, English imageability norms were sourced from Cortese and Fugett (2004) and Schock et al. (2012), with age of acquisition and frequency norms from Kuperman et al. (2012), and reaction times from Keuleers et al (2012).

A second version of the FaCE was developed for both French and English (version B). The linguistic parameters described above were again used to check for cross-linguistic similarity, as well as similarity between the stimuli on Version B to Version A. In all versions of FaCE, preference was for words that could be directly translated, as long as their psycholinguistic norms were similar. Care was also taken to choose semantically unrelated items for the word recall tasks, and to select words unrelated to the category of the semantic fluency task preceding the delayed recall task so as to minimize possible priming effects in delayed recall (e.g. since the semantic fluency asked participants to name *fruits and vegetables*, if the word list contained stimuli from this category then delayed recall might be facilitated as it occurred later in the test sequence) (Ruiz et al., 2018). Stimuli chosen for the word recall memory tests were uninflected content words with high imageabilty (Desrochers & Thompson (2009). As shown in Table 1, the language stimuli comparisons show no large differences between the French and English stimuli, nor between the stimuli on the two versions of the test.

Table 1: Average psycholinguistic norms for word list recall stimuli (version A and B)

	Imageability $\bar{x}$	AoA $\bar{x}$	Freq. $\bar{x}$	RT $\bar{x}$	Syll. ∑	Phon n ∑
Version A: French	6.7	4.78	33.7	633ms	11	28
Version B: French	6.14	6.65	34.64	633.58	12	27
Version A: English	6.4	5.2	42.23	536ms	11	28
Version B: English	6.11	5.77	46.90	534.06	12	27

1. Imageability $\bar{x}$ = average word ratings, Likert scale 0-7
2. AoA $\bar{x}$ = average age of acquisition
3. Freq. $\bar{x}$ = average frequency of word per million words
4. RT $\bar{x}$ = average reaction time in lexical decision experiments
5. Syll. ∑ = sum of syllables in the word list to be recalled
6. Phon n ∑ = sum of phonemes in the word list to be recalled

Because word frequency is the strongest predictor of lexical access accounting for up to 40% of the variance in reaction time experiments (Brysbaert & New, 2009; Brysbaert, Mandera, & Keuleers, 2017; Ferrand et al, 2010: 489), this was the most important variable to control for, and the comparisons in table 1 show that while the English versions had slightly higher word frequency, and faster mean reaction times, the differences are within a potentially negligible range in terms of cognitive demands cross-linguistically and in the two versions of FaCE. The different versions of FaCE are similar in the syllable lengths and

number phonemes in each word recall list, as well as having similar overall imageability and age of acquisition norms.

Beyond list recall subtests within FaCE, the semantic fluency task was examined from a psycholinguistic perspective. For semantic fluency, FRUITS and VEGETABLES was included in version A and for version B of the FaCE, the category of ANIMALS was chosen. Along with FRUITS and VEGETABLES, the ANIMALS category is the most common category in used in semantic fluency tests (Schwartz et al, 2003) and is considered diagnostically comparable in established cognitive evaluation tests such as the Repeatable Battery for the Assessment of Neuropsychological Status (RBANS) (Randolph et al., 1998) and the Cambridge Semantic Test Battery (Adlam, Patternson & Hodges, 2010). Animal naming norms per minute based on large participants pools vary but reported averages range from 16.9 (Rosselli et al, 2002), to 18.1 (Duff et al., 2004), to 26.35 and 25.65 for French and English speakers respectively (Roberts & Le Dorze, 1997: 423). Similar norms have recently been reported for animal naming by French speakers in Quebec (St-Hilaire, Simard & Bherer, 2016). Furthermore, the ANIMAL category in semantic fluency tests has been shown to be similar cross-linguistically, with Roberts and Le Dorze (1997) finding that French and English speakers did not differ on the number of animals named and Acevedo et al. (2000) report that animal naming fluency does not show marked variation by language background gender.

References

Acevedo, A., Loewenstein, D. A., Barker, W. W., Harwood, D. G., Luis, C., Bravo, M., ... & Duara, R. (2000). Category fluency test: normative data for English-and Spanish-speaking elderly. *Journal of the International Neuropsychological Society,* 6(7), 760-769.

Adlam, A. L. R., Patterson, K., Bozeat, S., & Hodges, J. R. (2010). The Cambridge Semantic Memory Test Battery: Detection of semantic deficits in semantic dementia and Alzheimer's disease. *Neurocase*, 16(3), 193-207.

Brysbaert, M., & New, B. (2009). Moving beyond Kučera and Francis: A critical evaluation of current word frequency norms and the introduction of a new and improved word frequency measure for American English. *Behavior Research Methods*, 41(4), 977-990.

Brysbaert, M., Mandera, P., & Keuleers, E. (2017). The word frequency effect in word processing: An updated review. *Current Directions in Psychological Science*, DOI: 0963721417727521.

Cortese, M., & Fugett, A. (2004). Imageability ratings for 3,000 monosyllabic words. *Behavior Research Methods, 36*(3), 384-387.

Desrochers, A., & Thompson, G. L. (2009). Subjective frequency and imageability ratings for 3,600 French nouns. *Behavior Research Methods*, 41(2), 546-557.

Duff, K., Schoenberg, M. R., Patton, D., Mold, J., Scott, J. G., & Adams, R. L. (2004). Predicting change with the RBANS in a community dwelling elderly sample. *Journal of the International Neuropsychological* Society, 10(6), 828-834.

Ferrand, L., Bonin, P., Méot, A., Augustinova, M., New, B., Pallier, C., & Brysbaert, M. (2008). Age-of-acquisition and subjective frequency estimates for all generally known monosyllabic French words and their relation with other psycholinguistic variables. *Behavior Research Methods*, *40*(4), 1049-1054.

Ferrand, L., New, B., Brysbaert, M., Keuleers, E., Bonin, P., Méot, A., Augustinova, M., & Pallier, C. (2010). The French Lexicon Project: Lexical decision data for 38,840 French words and 38,840 pseudowords. *Behavior Research Methods, 42,* 488-496.

Keuleers, E., Lacey, P., Rastle, K., & Brysbaert, M. (2012). The British Lexicon Project: Lexical decision data for 28,730 monosyllabic and disyllabic English words. *Behavior Research Methods*, *44*(1), 287-304.

Kuperman, V., Stadthagen-Gonzalez, H., & Brysbaert, M. (2012). Age-of-acquisition ratings for 30,000 English words. *Behavior Research Methods*, *44*(4), 978-990.

Randolph, C. (1998). Repeatable Battery for the Assessment of Neuropsychological Status (RBANS): test manual.

Roberts, P. M., & Le Dorze, G. (1997). Semantic organization, strategy use, and productivity in bilingual semantic verbal fluency. *Brain and Language*, 59(3), 412-449.

Rosselli, M., Ardila, A., Salvatierra, J., Marquez, M., Luis, M., & Weeks, V. (2002). A cross-linguistic comparison of verbal fluency tests. *International Journal of Neuroscience*, *112*(6), 759-776.

Ruiz, J. C., Soler, M. J., Dasí, C., Fuentes, I., & Tomás, P. (2018). The effect of associative strength on semantic priming in schizophrenia. *Psychiatry research*, 259, 1-6.

Schock, J., Cortese, M., & Khanna, M. (2012). Imageability estimates for 3,000 disyllabic words. *Behavior Research Methods*, *44*(2), 374-379.

Schwartz, S., Baldo, J., Graves, R. E., & Brugger, P. (2003). Pervasive influence of semantics in letter and category fluency: A multidimensional approach. *Brain and language*, 87(3), 400-411.

St-Hilaire, A., Hudon, C., Vallet, G. T., Bherer, L., Lussier, M., Gagnon, J. F., ... & Macoir, J. (2016). Normative data for phonemic and semantic verbal fluency test in the adult French–Quebec population and validation study in Alzheimer's disease and depression. *The Clinical Neuropsychologist*, 30(7), 1126-1150.
