# Supplementary material for: The Fast Cognitive Evaluation (FaCE): a screening tool to detect cognitive impairment in patients with cancer
Source: BMC Cancer. 2023 Jan 9;23:35. doi: 10.1186/s12885-022-10470-1 (PMC9830916; doi:10.1186/s12885-022-10470-1)
Supplement: Supplementary file 4 — Additional file 4. [file 12885_2022_10470_MOESM4_ESM.pdf]

Appendix 4

The Fast Cognitive Evaluation (FaCE)

INSTRUCTIONS FOR ADMINISTRATION

The FaCE questionnaire is an instrument conceived to quickly assess cancer patients' cognitive functions and follow their evolution. It assesses attention, memory, visuoconstruction and executive functions. It is printed on both sides of a single page. It is scored from 0 to 100% and usually takes 5 minutes to complete.

Required Materials:

Pens/Pencils (different types/sizes and colours)
Two stopwatches (or one stopwatch with the ability to perform two simultaneous timings)
One testing sheet (double-sided)
A calm room

1- General Information:

Before beginning the test, complete the general information in the document's first section.

- **ID**: the individual's identification number

Write down the individual's name and/or the file number if it is a clinical follow-up, or their identification number as assigned if it is a research project.

- **Education**: number of completed years.

Ask: *"Until now, how many years of school have you completed?"*
If the person struggles with counting the number of years of education they have completed, add them up by asking the highest education level reached and help them calculate the total using the following data: completed elementary school = 6 years; completed high school = 5 years; completed CEGEP and/or university: depends on the program studied (ask for additional details). Write down the number of years of education completed.

- **Date of Birth**

Ask for the individual's date of birth and enter it in the day/month/year format (DD/MM/YYYY).
Ask: *"What is your date of birth?"*

- **Dominant Hand**

Document the individual's dominant hand. Ask: "*Which hand is your dominant one?"*
Circle "Left" if they are left-handed, and "Right", if they are right-handed.

- **Sex**

Ask the person to indicate their sex by saying *"What is your sex?"* and circle the letter corresponding to their sex:
F, if she's a female,
M, if he's a male, or

- **Date**

Write down the date during which the test is administered (day/month/year: DD/MM/YYYY).

2- Orientation:

While not being part of the test itself, this section will act as a warm-up exercise.
Say *"We will start by a practice question that will not contribute to your test's final score. What is the date today?"*
If the given answer is incomplete, ask for precisions: *"Tell me today's year, month, date and the exact day."*

Then, ask: *"What season are we currently in?"*
Finally, ask information on the location: *"Tell me the name of the city we currently are in?"*
For each of the elements (date, month, year, day, season, city) check the corresponding box if the answer is correct.

<u>Beginning of the Test</u>

Inform the person that the test will now begin and that you will start the stopwatch.
Say: *"The test will now begin. I will ask you questions to which you will try to answer to the best of your ability. Please pay attention. If anything escapes you, do not hesitate to ask me questions. I will now start the stopwatch."*

Start the first stopwatch.

- ***Before beginning the next part, the <u>first</u> stopwatch must be started because the test begins now.***

3- Working Memory:

Inform the individual that you will read out loud a list 7 words and that they have to pay attention because they will have to repeat them once you are done. Make sure that they clearly understand the assignment before proceeding.

- **1st Try**

Read the 7-word list, clearly and audibly, at the rate of one word per second. If the individual repeats the correct word, check the box that corresponds to this word.
To inform the subject, say: *"I will read a wordlist that you will have to remember. Pay close attention. When I will be done, I want you to tell me as many words as possible that you remember from that list, in any given order".*

- **2nd Try**

After giving out the necessary instructions and checking the box corresponding to the words that the subject recalled during their first try, tell them: *"Now, I will read the same wordlist a second time. Try to remember and tell me as many words as you can from that list, even the ones that you recalled during your first try, in any given order"*. When the subject has completed their first try, no matter how many words were recalled, read the 7-word list again at the same rate of one word per second.
When the second try is done, inform the subject that he will have to remember these words because he will have to tell them again at the very end of the test. "*Try to remember the words from the list. I will ask you to repeat them to me at the end of the test.*"

4- Executive and Visuospatial Functions:

Show the person different kinds of pencils, so that they can choose the one that suits them best. Flip the document and show them the two sections that are on the other side.

- **1st Section: Alphanumeric Trajectory ('trail test')**

Inform the individual that they have to connect the letters and numbers on the page.
Tell them the instructions while pointing the appropriate spots on the document: *"I want you to draw a line without lifting the pencil, alternating from a number to a letter, while respecting the ascending numerical order and the alphabetical order. Start here (indicate 1) and draw the line to the letter A, then to the 2, and so on. End here (indicate the H)".*
Allow the individual to perform the activity. If they connect every part of the trajectory in the proper order, then the task was successfully completed. Check "Yes" on the first page.

© Bruno Gagnon 2016

If the individual makes 1 or more mistakes, then the task was not successfully completed. Check "No" on the first page.

- **2nd Section: 3D Cube Drawing**

Ask the subject to reproduce the presented cube. Say: *"I want you to copy this drawing in the most accurate way possible"*, while pointing at the cube model.

5- Attention

Ask the subject to subtract '7' from '101' and to continue to subtract '7' from the result of the first subtraction, until they are asked to stop. Make sure that he clearly understands the assignment.
Give out the following instructions: *"Now I want you to calculate 101 minus 7, and then, continue to subtract 7 from your answer, until I ask you to stop"*. If necessary, you can repeat the instructions.
Once the individual has completed 5 subtractions, ask them to stop.
If the FaCE questionnaire is to be administered during a future visit, perform the previous steps starting the subtractions with '103' instead of '101'.
Check the appropriate box for each result of the subtractions performed. If the individual fails the first subtraction or a subsequent subtraction, do not interrupt him and write down in the "Other Results" section the result obtained. The individual should continue to subtract '7' from the result even if it is wrong, and so on. Then, indicate the number of **sequential/successive** successful subtractions.

6- Language and Speed of Thought (verbal fluency)

Inform the individual that when you give them the signal, they will have to name as many fruits and vegetables as possible in a minute, in any given order. Make sure that they clearly understand the assignment: *"I want you to name as many different fruits and vegetables as possible. Do not name varieties of the same fruit or vegetable, for example, green grapes and red grapes. I will ask you to stop when one minute has passed. Are you ready? You can start now."*
Give the individual the signal to start and start the <u>second</u> stopwatch. Stop it once one minute has passed.
Write down the names of the fruits and vegetables enunciated by the individual or simply write down a check mark for every word named. If the same word is repeated, it will only be counted once. If varieties of the same fruit or vegetable are named (example: red grapes, green grapes), this will only count for a single fruit or vegetable.
When a minute has passed, inform the individual that the assignment is complete and stop the second stopwatch.

7- Delayed Recall

Ask the subject to name the words from the 7-word list stated at the beginning of the test.
"Earlier, I read out loud a wordlist that I asked you to remember. Now, I want you to name every word that you remember from that list."

<u>End of Test</u>

- ***The test ends here, stop the <u>first</u> stopwatch.***

The time on the first stopwatch corresponds to the test duration. Write down the duration of the test in minutes and seconds in the section "Test Duration" at the bottom of the first page.
Write down initials of the professional who administered the FaCE questionnaire in the section 'Administered by'.

© Bruno Gagnon 2016

Test Scoring

To calculate the individual's test final score, add up every point obtained in each task. This score corresponds to the individual's total and final score to the test. To find out the percentage corresponding to the individual's final score, please refer to the table which runs along on the righthand side of the first page of the questionnaire. For a more precise percentage, please refer to the table below. Please note that you will not obtain the correct percentage by dividing the number of points obtained by the total of 27 points.

Score	Percentage (%)		Score	Percentage (%)
0	0		14	44.63
1	13.61		15	46.26
2	20.98		16	47.98
3	25.14		17	49.81
4	28.06		18	51.80
5	30.36		19	53.94
6	32.30		20	56.27
7	34.03		21	58.82
8	35.62		22	61.67
9	37.14		23	65.00
10	38.62		24	69.17
11	40.09		25	74.92
12	41.57		26	84.45
13	43.07		27	100

© Bruno Gagnon 2016
